# Isolation and Characterization of Two Perfluorobutane Sulfonamide (FBSA)-Degrading Bacterial Strains, *Neobacillus* sp. LH-1 and *Glutamicibacter* sp. BO-1, from Estuarine and Marine Sediments

**DOI:** 10.3390/toxics14070564

**Published:** 2026-06-27

**Authors:** Chenhe Zhao, Mengjin Feng, Kairui Wang, Yvyan Gao, Jiasong Zhao, Shuyan Zhao

**Affiliations:** Key Laboratory of Industrial Ecology and Environmental Engineering (Ministry of Education), School of Chemical Engineering, Ocean and Life Sciences, Dalian University of Technology, Panjin 124221, China; 2156535046@mail.dlut.edu.cn (C.Z.); 20233271165@mail.dlut.edu.cn (M.F.); 2576382869@mail.dlut.edu.cn (K.W.); gaoyuyan919@mail.dlut.edu.cn (Y.G.); 20233271129@mail.dlut.edu.cn (J.Z.)

**Keywords:** perfluorobutane sulfonamide (FBSA), *Neobacillus*, *Glutamicibacter*, biodegradation, genetic framework analysis

## Abstract

Perfluorobutane sulfonamide (FBSA), an emerging short-chain perfluorooctanesulfonate (PFOS) alternative used in semiconductor manufacturing and fire suppression, has been detected in environmental matrices and poses environmental risks via industrial emissions and product leaching. However, the microbial degradation characteristics of FBSA are still unclear. In this study, two FBSA-transforming bacterial strains (designated LH-1 and BO-1) were isolated from the contaminated sediments of the Liaohe Estuary and the Bohai Sea, northeastern China. Based on 16S rDNA gene sequence analysis, strain LH-1 showed 99.66% sequence similarity with *Neobacillus cucumis* C7-N-8-8, while strain BO-1 showed 100% similarity with *Glutamicibacter nicotianae* OTC-16. Genomic analysis identified key degradation-related genes, including oxidoreductases, hydrolases, and genes involved in chloroalkane (LH-1) or fluorobenzoate (BO-1) degradation pathways, providing genetic evidence that supported their FBSA biotransformation potential. After 5 days of incubation with 133.8 nmol/mL FBSA, LH-1 and BO-1 removed 8.78% and 11.37% of FBSA, respectively. Perfluorobutanesulfonic acid (PFBS), perfluorobutanoic acid (PFBA), and perfluoropropionic acid (PFPrA) were detected as biodegradation products, with PFBS and PFPrA as the main products in strains LH-1 and BO-1, respectively. Genome annotation revealed candidate genes associated with deamination, oxidation, desulfonation, decarboxylation, and defluorination, as well as strain-specific enrichment of chloroalkane degradation genes in LH-1 and fluorobenzoate degradation genes in BO-1. Neither strain showed detectable degradation of perfluorooctanoic acid (PFOA) or PFOS, suggesting an apparent preference for the sulfonamide precursor FBSA over terminal perfluoroalkyl acids (PFAAs). This study provides the first genomic and metabolic insights into FBSA biotransformation by coastal sediment bacteria and improves our understanding of the environmental fate of sulfonamide-based PFAS precursors.

## 1. Introduction

Per- and polyfluoroalkyl substances (PFAS), valued for their exceptional thermal and chemical stability and unique amphiphilic properties [1,2], have been widely used in the manufacturing of aqueous film-forming foams (AFFF), textile coatings, and electroplating processes, leading to their environmental contamination [3]. Due to the bioaccumulation, persistence, and toxicity of perfluorooctanesulfonic acid (PFOS) and perfluorooctanoic acid (PFOA), they have been restricted under the Stockholm Convention on Persistent Organic Pollutants (POPs), promoting the introduction of alternative compounds [2]. Among these substitutes, short-chain perfluoroalkane sulfonamides (FASAs) represent a major class of PFOS replacements, with perfluorobutane sulfonamide (FBSA) serving as a key alternative and a critical intermediate in the synthesis of modern short-chain fluorosurfactants [4].

FBSA is a fluorinated C4 compound containing a sulfonamide group (-SO_2_NH_2_), and it is used as a C4 sulfonyl fluoride derivative in semiconductor manufacturing [4]. It has higher water solubility than its longer-chain analogs and exhibits significant environmental mobility [5]. This distinct chemical structure allows it to easily bypass conventional wastewater treatment plants and accumulate in aquatic ecosystems [6,7]. FBSA has been frequently detected in the environment and biota, with average concentrations of 1.66 ng/L in seawater, 4.31 ng/g dw in sediment, and 27.05 ng/g ww in marine biota from Liaodong Bay [8]. Exposure to FBSA and its environmental transformation products poses severe risks to human and ecological health. Recent toxicological research indicates that FBSA can impede vital biological processes and exhibits higher endocrine-disrupting potential and systemic toxicity than many traditional pollutants [9,10]. Furthermore, the continuous release of FBSA into the natural environment can intensify the global burden of fluorinated contaminants and threaten the ecological balance of terminal sinks, such as estuarine and marine sediments [11]. Thus, the accumulation of FBSA in sediments exerts selective pressure on microbiota, highlighting its importance for identifying tolerant degraders.

As a representative of short-chain FASAs, FBSA belongs to a broader class of PFAS precursors that exhibit diverse environmental behaviors. FASAs, a major subclass of PFAS precursors, include compounds with varying perfluorinated carbon chain lengths, such as C4 (FBSA), C6 (perfluorohexane sulfonamide, FHxSA), and C8 (perfluorooctane sulfonamide, PFOSA) [12]. Previous studies have extensively investigated the microbial degradation of longer-chain FASAs containing an eight-carbon perfluorinated chain (C8), including both N-alkyl-substituted compounds, such as N-ethylperfluorooctane sulfonamide (EtFOSA) and N-ethylperfluorooctane sulfonamidoacetic acid (EtFOSAA), and perfluorooctane sulfonamide (PFOSA). Their microbial degradation in soils has been relatively well characterized, with some persistent sulfonamidoacetic acid intermediates exhibiting half-lives of up to 335 days [13]. An *Acinetobacter* sp. M strain has been shown to degrade PFOSA via extracellular enzymes [14]. Similarly, C6 sulfonamido precursors can be converted to perfluorohexanesulfonate (PFHxS) by nitrifying microorganisms in groundwater systems [15].

To date, several bacterial consortia and pure cultures capable of biotransforming PFAS have been reported, including *Pseudomonas* sp. [16], *Acidimicrobium* sp. [17], and *Gordonia* sp. [18,19]. Mechanistically, the microbial transformation of PFAS is predominantly mediated by specific metabolic pathways. These pathways target structurally vulnerable sites in molecules, mainly functional headgroups, thereby converting parent compounds into partially fluorinated intermediates. Furthermore, under anaerobic conditions, certain specialized bacteria can perform reductive defluorination to cleave the recalcitrant carbon-fluorine (C–F) bonds, ultimately releasing inorganic fluoride ions [20]. However, despite these advances in understanding the degradation of longer-chain FASAs, the biodegradation of short-chain analogs like FBSA remains poorly characterized, primarily due to formidable thermodynamic challenges. The robust C–F shield renders the perfluorinated backbone resistant to typical enzymatic attacks [20], while the highly stable sulfonamide sulfur-nitrogen (S–N) linkage further exacerbates the recalcitrance of FASAs [12,21]. Therefore, there is an urgent need to explore novel, highly tolerant functional microorganisms with enhanced FBSA degradation capabilities in chronically polluted environments.

The Liaohe Estuary, located in Liaodong Bay within the semi-enclosed and highly industrialized Bohai Sea, represents a typical sink for PFAS contamination. The semi-enclosed Bohai Sea, characterized by limited water exchange and intensive industrial activities in its surrounding regions, is highly vulnerable to PFAS contamination from industrial and firefighting sources [8]. Two major fluorochemical production bases are distributed around this region, and total PFAS concentrations in inflowing rivers have been reported to reach 660 μg/L [22]. The Liaohe Estuary and the Bohai Sea are recognized as important sinks for fluorinated pollutants and may impose selective pressure on resident microbial communities [8,23,24,25,26]. Nevertheless, the environmental fate and biodegradation potential of emerging short-chain alternatives such as FBSA remain largely unexplored in this ecosystem.

This study therefore aimed to isolate and characterize FBSA-degrading bacterial strains from the contaminated sediments of the Liaohe Estuary and the Bohai Sea. Furthermore, we employed whole-genome sequencing (WGS) to identify genetic determinants and putative catabolic pathways governing FBSA biodegradation, and systematically evaluated the substrate specificity of the isolates toward legacy PFOS and PFOA. This study will provide new insights into the microbial fate of sulfonamide-based PFAS precursors in coastal environments.

## 2. Materials and Methods

### 2.1. Reagents and Materials

FBSA (98%), PFOS (98%), PFOA (98%), trifluoroacetic acid (TFA, 99%), perfluoropropanoic acid (PFPrA, 97%), and perfluorobutanoic acid (PFBA, 98%) were purchased from Shanghai Macklin Biochemical Technology Co., Ltd. (Shanghai, China). Perfluoropentanoic acid (PFPeA, 97%), perfluoroheptanoic acid (PFHpA, 98%), PFHxS (98%), and perfluorobutanesulfonate (PFBS, 98%) were obtained from J&K Scientific Ltd. (Beijing, China). Perfluorohexanoic acid (PFHxA, 98%) was provided by Matrix Scientific (Columbia, SC, USA). HPLC-grade methanol (99.9%) and analytical-grade (AR) ethanol were purchased from Dalian Bonno Biochemical Reagents Co., Ltd. (Dalian, China). Milli-Q water (18.2 MΩ·cm, TANKPE060) was used throughout the experiments.

### 2.2. Environmental Sampling, Enrichmen55t, and Isolation

**Figure 1:**
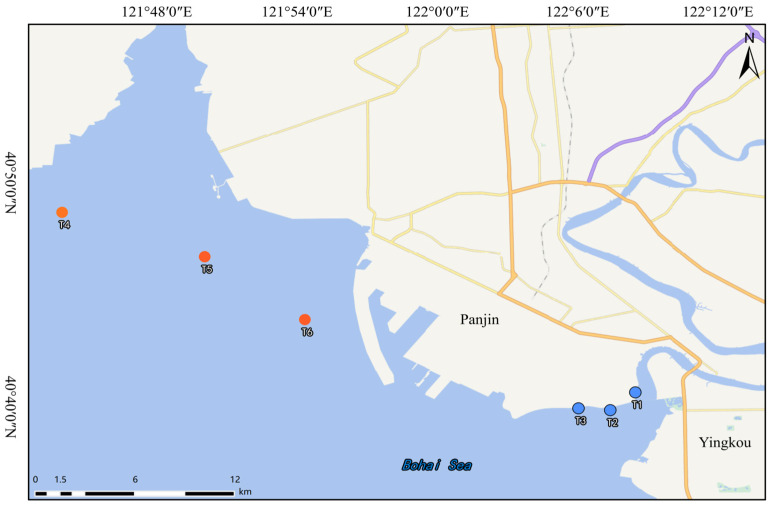
Sampling area and sediment sampling points in the Liaohe Estuary and the Bohai Sea. The blue circles represent the sampling points for Liaohe Estuary sediments, and the orange circles represent the sampling points for Bohai Sea marine sediments.

Our previous study revealed that FBSA was detectable in this region (sediments, 4.31 ng/g dw) [8], making it suitable for isolating FBSA-degrading bacteria. As shown in Figure 1 and Table S1, sediment samples were collected from the highly industrialized Liaohe Estuary and the adjacent Bohai Sea, China. Triplicate samples were collected at each location and homogenized to form a composite sample. Samples (top 5 cm layer) were collected using a stainless-steel grab sampler and stored in 1 L polypropylene (PP) bottles. To initiate the selective enrichment process, 5.0 g of the homogenized sediment from each site was inoculated into 250 mL Erlenmeyer flasks containing 100 mL of enrichment broth consisting of mineral salt medium (MSM) supplemented with 1.0 g/L yeast extract. The medium was initially spiked with 33.44 nmol/mL of FBSA as the target stressor. The cultures were incubated aerobically in a rotary shaker at 30 °C and 150 rpm in the dark to prevent any potential photodegradation of the fluorinated compounds.

A stepwise acclimatization strategy was implemented to progressively enhance microbial tolerance to fluorinated compound toxicity. Every 5 days, a 5.0 mL aliquot (5% *v*/*v*) of the active suspension was transferred into fresh enrichment medium containing an incrementally elevated FBSA concentration (increasing by 33.44 nmol/mL per cycle, up to a maximum of 133.8 nmol/mL). Subsequently, to minimize the proliferation of non-specific heterotrophs reliant on easily degradable carbon sources, the highly enriched consortia were subcultured into strictly inorganic MSM spiked with a constant FBSA concentration of 133.8 nmol/mL. This stringent subculturing process was repeated for four consecutive cycles (20 days in total) under identical optimal incubation conditions.

For the isolation of pure bacterial strains, 1.0 mL of the fully acclimatized culture was serially diluted (10^−1^ to 10^−6^) using sterile physiological saline (0.85% NaCl). Aliquots (100 μL) from the 10^−2^, 10^−4^, and 10^−6^ dilutions were uniformly spread onto solid MSM agar plates supplemented with 133.8 nmol/mL FBSA. Following a 5-day incubation at 30 °C, morphologically distinct bacterial colonies exhibiting robust growth were selected. These colonies were repeatedly streaked onto fresh solid plates for a minimum of four successive rounds to ensure the complete acquisition of axenic (pure) cultures. Ultimately, two strains with distinct morphological and ecological characteristics, designated LH-1 from the Liaohe Estuary and BO-1 from the Bohai Sea sediments, were successfully isolated. The purified strains were cryopreserved in 20% (*v*/*v*) sterile glycerol at −20 °C for subsequent genomic identification and biodegradation assays.

### 2.3. Physiological Tolerance and Morphological Characterization

To evaluate the growth dynamics and physiological tolerance of strains LH-1 and BO-1 under PFAS stress, bacterial growth kinetics were systematically monitored. Seed cultures grown to the mid-logarithmic phase were harvested by centrifugation (5000 rpm, 5 min) and washed three times with 0.1 M phosphate-buffered saline (PBS, pH 7.4) to remove residual organic carbon. The washed bacterial suspensions were inoculated at 1.0% *v*/*v* into 250 mL Erlenmeyer flasks containing 100 mL of enrichment medium spiked with 133.8 nmol/mL FBSA. The cultures were incubated aerobically at 30 °C and 150 rpm. For the precise determination of the exponential growth phase, liquid aliquots were sampled every 2 h during the initial 12 h of incubation, followed by daily sampling over a 7-day period. At each time point, 200 μL of the culture broth was dispensed into a 96-well microplate, and the optical density at 600 nm (OD_600_) was measured using a multi-mode microplate reader (SpectraMax M5, Molecular Devices, San Jose, CA, USA). Uninoculated flasks containing identical media formulations served as abiotic controls, and all assays were performed in triplicate. The measured growth curves of LH-1 and BO-1 are shown in Figure S1.

For microscopic morphological characterization, bacterial cells cultivated in the FBSA-free enrichment broth (MSM supplemented with 1.0 g/L yeast extract) were harvested at the mid-logarithmic phase via centrifugation (5000 rpm, 3 min) and washed gently three times with PBS to remove the residual culture matrix. The cell pellets were subsequently fixed in a 2.5% *v*/*v* glutaraldehyde solution at 4 °C for 24 h to preserve the cellular architecture. Following primary fixation, the specimens were dehydrated through a sequential graded ethanol series (20%, 40%, 60%, 80%, and 100%, *v*/*v*), with a 10 min equilibration period per step. Finally, the fully dehydrated samples were sputter-coated with a thin layer of gold and observed under a scanning electron microscope (Nova Nano SEM 450, FEI Company, Hillsboro, OR, USA), with full instrumental parameters provided in the Supplementary Materials.

### 2.4. Taxonomic Identification and Whole-Genome Framework Analysis

The genomic DNA of the purified strains LH-1 and BO-1 was extracted using the Ezup Column Bacteria Genomic DNA Extraction Kit (Sangon Biotech, Shanghai, China). The 16S rDNA gene was amplified via polymerase chain reaction (PCR) utilizing the universal bacterial primers 27F (5′-AGTTTGATCMTGGCTCAG-3′) and 1492R (5′-GGTTACCTTGTTACGACTT-3′). The purified amplicons were subjected to standard Sanger sequencing, which was commercially performed by Sangon Biotech (Shanghai, China). The acquired contiguous sequences (approximately 1450 bp) were queried against the NCBI GenBank database using BLASTn (NCBI BLAST, 2.17.0). To precisely determine their evolutionary positions, a phylogenetic tree was constructed based on the neighbor-joining method using MEGA 12 software, with the topological robustness rigorously evaluated by bootstrap analysis utilizing 1000 replicates. The 16S rDNA gene sequences of strains LH-1 and BO-1 have been deposited in the NCBI GenBank database under the accession numbers PZ254056 and PZ250457, respectively.

A WGS framework analysis was performed on the two strains. Highly qualified genomic DNA samples were randomly fragmented using a Covaris ultrasonic processor (Covaris, Woburn, MA, USA). Sequencing libraries were constructed and subsequently sequenced on an Illumina platform to generate paired-end reads. To guarantee high downstream accuracy, the raw sequencing data were stringently filtered using the fastp bioinformatics tool. Specifically, adapter sequences, poly-G tails, reads with an “N” base content exceeding 10%, and low-quality reads (where >50% of bases possessed a quality score of Q ≤ 5) were removed to yield high-quality clean data [27]. Following de novo genome assembly, comprehensive genome component prediction and functional annotation were performed by mapping coding sequences against major biological databases, prominently including Gene Ontology (GO, https://geneontology.org/, accessed on 23 April 2026), the Kyoto Encyclopedia of Genes and Genomes (KEGG, https://www.kegg.jp/, accessed on 23 April 2026) and Cluster of Orthologous Groups (COG, https://www.ncbi.nlm.nih.gov/research/cog-project/, accessed on 23 April 2026). This customized genomic framework was specifically tailored to mine putative functional gene clusters encoding amidases, dehalogenases, and desulfonases.

### 2.5. Biodegradation Assays and Substrate Specificity of Isolated Strains

To establish standardized inocula, individual colonies of strains LH-1 and BO-1 were cultured in enrichment broth at 30 °C and 150 rpm until the mid-logarithmic phase (OD_600_ ≈ 0.4–0.5). The cells were then harvested via centrifugation (5000 rpm, 5 min) and washed three times with sterile 0.1 M phosphate-buffered saline (PBS, pH 7.4). The resulting pellets were resuspended in PBS to prepare standardized inocula.

The MSM was individually spiked with FBSA, PFOS, or PFOA at an initial concentration of 133.8 nmol/mL, serving as the sole added carbon and energy sources for the microorganisms. Subsequently, 0.1 mL of the standardized seed suspension was inoculated into respective assay systems. The cultures were incubated aerobically in a rotary shaker at 30 °C and 150 rpm. To preclude photodegradation, all experimental systems were protected from light during the incubation period. Abiotic controls (uninoculated MSM spiked with PFAS) and killed controls (MSM inoculated with heat-autoclaved bacterial suspensions) were included. All treatments and controls were performed in biological triplicate. After 5 days, the entire culture broth was collected for analyte extraction and mass spectrometric quantification.

### 2.6. Sample Extraction and UPLC-MS/MS Quantification

Briefly, 1.0 mL of the collected exposure broth was diluted to 50 mL with ultrapure water. Sample purification was performed using Cleanert HLB-SPE cartridges (500 mg/6 mL, Agela Technologies, Tianjin, China) [28]. The cartridges were pre-conditioned sequentially with 10 mL of methanol and 10 mL of ultrapure water. The diluted samples were loaded at a flow rate of one drop per second. After washing with 5 mL of ultrapure water and vacuum drying, the target analytes were eluted with 10 mL of methanol into 15 mL polypropylene (PP) centrifuge tubes. The eluents were concentrated to approximately 0.5 mL under a gentle stream of nitrogen, reconstituted to 1.0 mL with methanol and ultrapure water (1:1 *v*/*v* methanol/ultrapure water), filtered through a 0.22 μm polytetrafluoroethylene (PTFE) syringe filter, and transferred into amber autosampler vials for analysis.

Chromatographic separation and quantification were performed on a Waters XEVO-TQS UPLC-MS/MS system (Waters Corporation, Milford, MA, USA). Analytes were resolved using a BEH C18 analytical column (1.7 μm, 2.1 mm × 50 mm, Waters, Milford, MA, USA) equipped with a corresponding guard column, maintained at 38 °C. The mobile phases comprised 2 mmol/L aqueous ammonium acetate (Solvent A) and methanol (Solvent B) at a flow rate of 0.45 mL/min. The detailed gradient elution program is provided in Table S2. Mass spectrometric detection was operated in negative electrospray ionization (ESI−) mode using multiple reaction monitoring (MRM). The optimized key MS parameters included a capillary voltage of 2.2 kV, a desolvation temperature of 400 °C, a desolvation gas flow of 800 L/h, and a cone gas flow of 150 L/h. The specific MRM transitions, collision energies, and cone voltages for all target PFAS are summarized in Table S3.

### 2.7. Quality Assurance and Quality Control (QA/QC)

To systematically validate the feasibility and accuracy of the external standard quantification method, matrix spike-and-recovery tests were conducted. Target mixed standards were spiked into uninoculated blank biological matrices at a precisely defined theoretical concentration. The method detection limits (MDLs) and method quantification limits (MQLs) were determined at signal-to-noise ratios of 3:1 and 10:1, respectively, using blank matrices. The recoveries for all monitored PFAS in both LH-1 and BO-1 culture matrices ranged from 72% to 128%. The recoveries, MDLs, and MQLs of the analyzed PFAS are shown in Table S3.

### 2.8. Data and Statistical Analysis

The degradation efficiency (E, %) was calculated as:

(1) $$E\left(\%\right)=\frac{C_{0d}\;-{\;C}_{5d}}{C_{0d}}\;\times \;100$$

where *C*_0*d*_ is the concentration (nmol/mL) of the parent compound in the abiotic control on day 0, and *C*_5*d*_ is the residual concentration (nmol/mL) in the biotic treatments on day 5.

The molar mass balance (MB, %) was calculated using Equation (2) to track the conversion of parent compounds into products:

(2) $$\mathrm{MB}\left(\%\right)=\frac{M_{\mathrm{residual},\mathrm{parent}}+\sum M_{\mathrm{metabolites}}}{M_{\mathrm{initial},\mathrm{control}}}\;\times \;100$$

where $M_{\mathrm{residual},\mathrm{parent}}$ represents the molar concentration of the residual parent compound (in nmol/mL) after 5 days of degradation. $\sum M_{\mathrm{metabolites}}$ refers to the molar concentrations (in nmol/mL) of various degradation metabolites (e.g., PFBS, FBSA, PFPrA). $M_{\mathrm{initial},\mathrm{control}}$ denotes the initial molar concentration of the parent compound spiked into the system at the start of the experiment.

Data processing, visualization, and statistical analyses were performed using Origin Pro 2024 (OriginLab, Version 10.3.0.197, USA). Independent Student’s *t*-tests were performed using IBM SPSS Statistics 27 (IBM Corp., Version 32.0.0.0, Armonk, NY, USA) to compare the biotransformation capacities and metabolite accumulation profiles between strains LH-1 and BO-1, after confirming homogeneity of variances. Statistical significance was set at *p* < 0.05.

## 3. Results and Discussion

### 3.1. Isolation and Identification of FBSA-Degrading Strains

**Figure 2:**
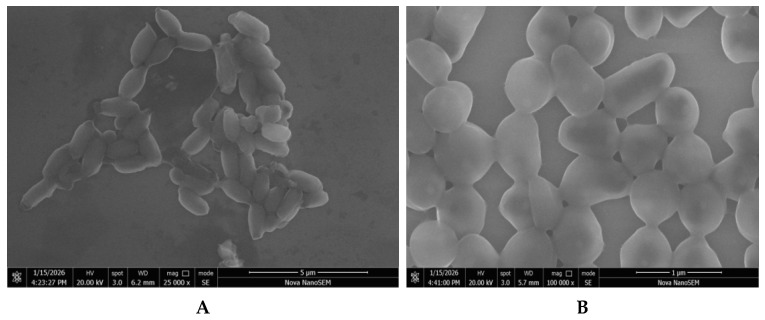
SEM micrographs of strains LH-1 (**A**) and BO-1 (**B**).

**Figure 3:**
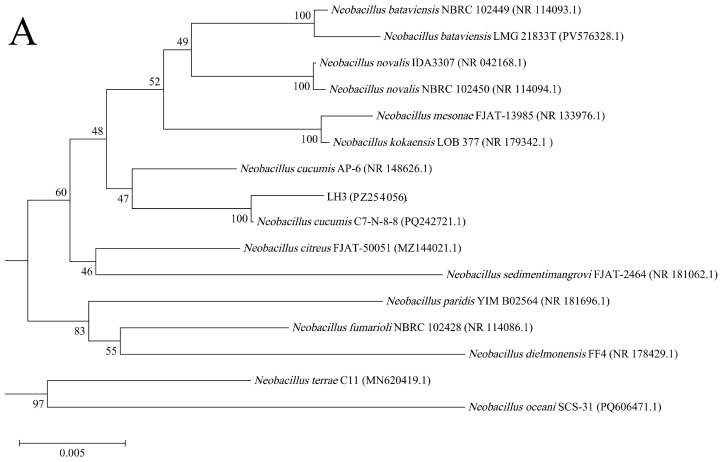

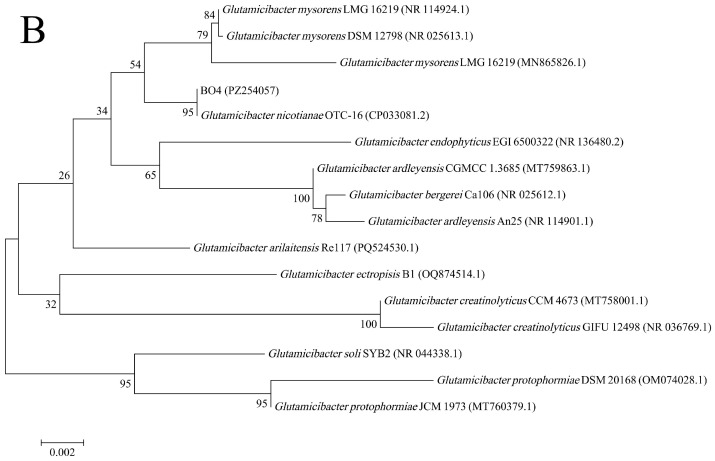
Phylogenetic trees of strains LH-1 (**A**) and BO-1 (**B**) based on 16S rDNA gene sequences.

The purified strains LH-1 and BO-1 were isolated from sediment enrichment cultures and characterized by SEM and 16S rDNA sequencing. SEM observation (Figure 2) showed that both isolates were rod-shaped with smooth cell surfaces. Strain LH-1 measured approximately 1.0–1.7 μm in length, whereas strain BO-1 measured about 1.0 to 1.5 μm. The 16S rDNA sequence of LH-1 was 1473 bp long. In the neighbor-joining tree (Figure 3), LH-1 clustered with *Neobacillus cucumis* C7-N-8-8, with a bootstrap value of 100% and a sequence similarity of 99.66%, supporting its assignment to the genus *Neobacillus*. The 16S rDNA sequence of BO-1 was 1452 bp long and clustered with *Glutamicibacter nicotianae* OTC-16, with a bootstrap value of 95% and a sequence similarity of 100%, supporting its assignment to the genus *Glutamicibacter*.

Previous investigations in this region revealed that Proteobacteria and Bacteroidetes dominated the sediment bacterial communities, whereas Firmicutes and Actinobacteria were much less abundant [29,30]. The isolates *Neobacillus* (Firmicutes) and *Glutamicibacter* (Actinobacteria) may thus represent low-abundance yet functionally significant taxa, which are often overlooked by conventional high-throughput sequencing but retrievable through selective enrichment and pure-culture isolation.

*Neobacillus* and *Glutamicibacter* are both genera with documented capacities for pollutant degradation, though their role in PFAS biotransformation has not been reported. Species within the genus *Neobacillus* have been isolated from diverse environments and shown to degrade a range of organic contaminants. For example, two *Neobacillus* strains LXY-1^T^ and LXY-4^T^ were isolated from soil samples obtained from a heavy metal smelter in Guangxi. LXY-1^T^ and LXY-4^T^ were capable of removing heavy metals and toxic contaminants through adsorption, transformation, and degradation [31]. A *Neobacillus* strain NS-6, isolated from sandstone oil in the Ordos Basin, exhibited excellent urease-producing capacity and high efficiency in inducing calcium carbonate precipitation [32]. The genus *Glutamicibacter* has similarly been associated with the biodegradation of diverse pollutants. *Glutamicibacter* strain S2, isolated from untreated activated sludge, could efficiently biodegrade cephalexin [33]. A novel pyrene-degrading bacterium, *Glutamicibacter soli* ENR6, isolated from petroleum-contaminated soil, could utilize various PAHs as the sole carbon source, degrading 88.8% of 50 mg/L pyrene and 76.8% of 100 mg/L pyrene within 15 days [34].

To our knowledge, despite the documented pollutant-degrading capabilities of both genera, neither *Neobacillus* nor *Glutamicibacter* has previously been implicated in PFAS biotransformation. These isolates therefore expand the known taxonomic range of FBSA-transforming bacteria and suggest that the capacity for sulfonamide-based PFAS precursor transformation is more broadly distributed across environmental genera than previously recognized.

### 3.2. Genomic Features and Identification of Putative Degradation-Related Genes

**Figure 4:**
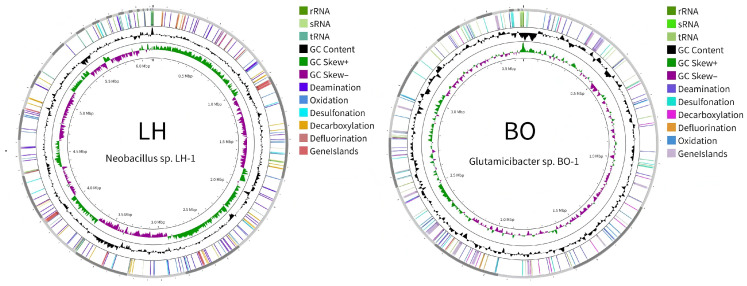
Graphical circular genome maps of *Neobacillus* sp. LH-1 (**left**) and *Glutamicibacter* sp. BO-1 (**right**). The outermost circle displays the scale in megabase pairs (Mbp). The second circle shows the distribution of functional genes involved in key metabolic processes (including deamination, desulfonation, decarboxylation, defluorination, and oxidation) and genomic features (rRNA, sRNA, tRNA, and genomic islands). The third circle shows the G + C mol% content plot. The innermost circle shows GC skew, with purple indicating negative values and green indicating positive values.

Whole-genome sequencing statistics of strains LH-1 and BO-1 are summarized in Figure 4, with detailed data listed in Table S4. Strain LH-1 had a genome size of 6.12 Mbp, 6385 predicted genes, and a GC content of 38.92%. Strain BO-1 had a genome size of 3.66 Mbp, 3390 predicted genes, and a GC content of 61.97%.

**Figure 5:**
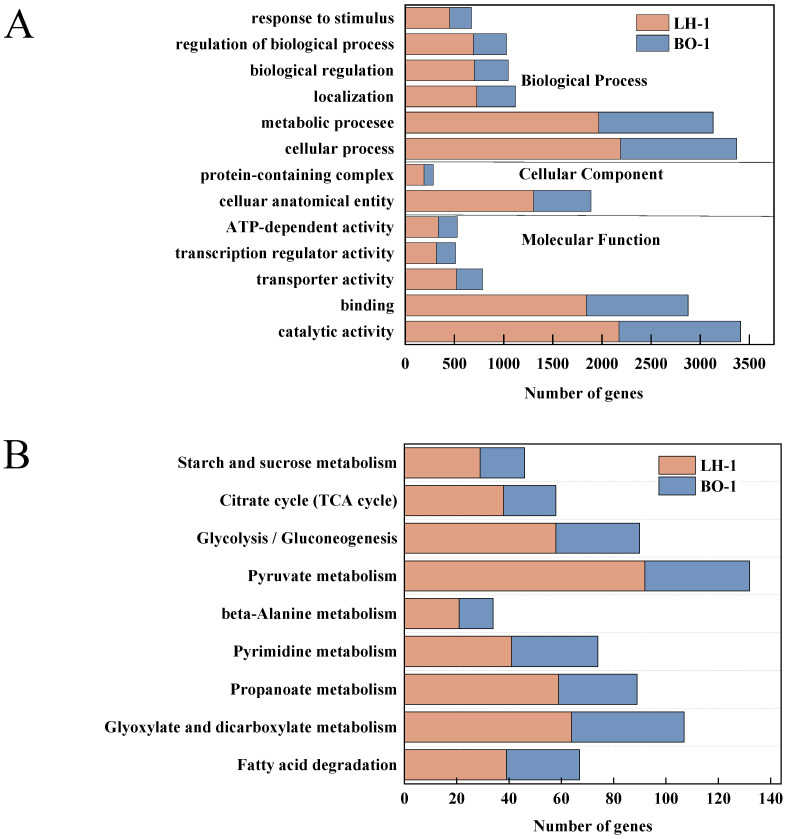
Functional annotation of genes based on GO and KEGG analyses. (**A**) GO functional classification of annotated genes into Biological Process, Cellular Component, and Molecular Function categories. (**B**) KEGG-based annotation of genes associated with key central carbon and energy metabolism pathways.

Functional annotation was performed against the GO, KEGG, and COG databases. GO functional annotation and KEGG pathway enrichment analysis of strains LH-1 and BO-1 are compared in Figure 5. Both strains showed a high proportion of genes associated with central carbon metabolism pathways, including pyruvate metabolism. These broad categories mainly indicate general metabolic capacity rather than FBSA-specific activity. KEGG annotation also suggested several differences that may be relevant to the distinct product patterns observed later in the degradation assay. Strain LH-1 contained 13 genes annotated in the chloroalkane or chloroalkene degradation pathway, whereas no fluorobenzoate-degradation genes were identified. In contrast, strain BO-1 contained 6 genes annotated in the fluorobenzoate degradation pathway, together with 9 genes annotated as dehalogenase-related genes.

The differential annotation patterns between the two strains are notable in the context of their distinct metabolite profiles. The presence of fluorobenzoate degradation genes exclusively in BO-1 is consistent with its capacity for more extensive chain shortening, as evidenced by the dominant accumulation of the C3 product PFPrA (Section 3.3). Fluorobenzoate degradation pathways typically involve dearomatization and subsequent carbon-chain truncation reactions [35], which may provide BO-1 with enzymatic machinery capable of processing the perfluorinated backbone beyond the initial desulfonamidation step. Although the typical substrates of these pathways (fluorobenzoates) differ structurally from perfluoroalkyl sulfonamides, the core reaction types (e.g., carbon–carbon bond cleavage leading to chain truncation) may still be mechanistically relevant. In contrast, the absence of fluorobenzoate-related genes in LH-1, coupled with its exclusive enrichment of chloroalkane and chloroalkene degradation genes, is consistent with the finding that this strain produces PFBS as the dominant transformation product, together with minor yields of PFBA and PFPrA. Chloroalkane degradation pathways primarily involve dehydrohalogenation and hydrolytic reactions that target halogen-substituted carbons [35], which likely act inefficiently on the fully fluorinated carbon backbone of PFBS, thereby limiting further breakdown into shorter-chain perfluorinated products.

The large pools of oxidoreductase and hydrolase genes in both strains likely play a significant role in FBSA biotransformation. In PFAS biodegradation studies, cytochrome P450 oxidoreductases and other oxidoreductases are known to mediate the initial oxidation of sulfonamide headgroups and the subsequent oxidation of perfluorinated intermediates [36,37]. *Gordonia* sp. strain NB4-1Y was reported to utilize oxidoreductase-mediated reactions in the biotransformation of 6:2 fluorotelomer sulfonamidoalkyl betaine, in which oxidation of the non-fluorinated portion preceded further chain shortening [19]. The extracellular enzyme system of *Acinetobacter* sp. M, which degrades PFOSA, was proposed to involve hydrolytic activity at both C–C and C–F bonds [14]. The abundant oxidoreductase and hydrolase gene repertoires identified in both LH-1 and BO-1 therefore provide a plausible enzymatic basis for initiating FBSA desulfonamidation and enabling subsequent downstream transformations. However, these annotations alone are not sufficient to confirm FBSA-degrading activity, and similar biochemical validation has been required for candidate genes in the PFAS biodegradation studies [38]. The genome data are therefore used to identify candidate functions, which are then cross-referenced against the metabolite profiles and the pathway hypothesis detailed in Section 3.3.

### 3.3. Degradation Characteristics and Biotransformation Pathways of FBSA

#### 3.3.1. Degradation Efficiency and Metabolite Profiling

**Figure 6:**
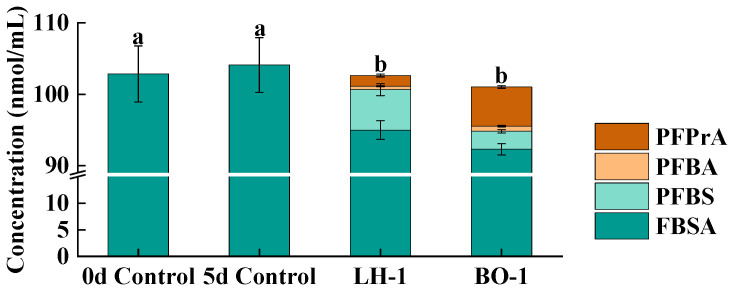
Concentrations of FBSA and its degradation products in different treatment groups. Data are presented as mean ± standard deviation (SD), *n* = 3. Different letters indicate significant differences (*p* < 0.05).

As shown in Figure 6, the abiotic control showed no significant change in FBSA concentration over the 5-day period (*p* > 0.05), indicating that non-biological loss, including volatilization, sorption, and abiotic degradation, was negligible under the tested conditions. In the biotic treatments, both strains significantly reduced FBSA concentrations compared with the abiotic control (*p* < 0.05). After 5 days of incubation, strain LH-1 achieved a FBSA removal efficiency of approximately 8.78%, while strain BO-1 achieved approximately 11.37%. These results demonstrate that both bacterial isolates are capable of metabolizing FBSA, with strain BO-1 exhibiting a slightly higher removal efficiency. This phenomenon contrasts with previous findings that freshwater sediment microbial communities collected from Taihu Lake exhibited significantly higher biodegradation efficiencies toward high-molecular-weight FASAs (e.g., SAmPAP diester) than marine sediment communities from False Creek, Vancouver, Canada [39,40]. These removal efficiencies are comparable to those reported in our previous studies on long-chain FASA biotransformation under short-term aerobic conditions. For example, our previous study isolated a strain, *Hyphomicrobium* sp. PF1, from soil near a fluorochemical plant, which utilized PFOSA and N-ethyl perfluorooctyl sulfonamide (N-EtFOSA) as the sole carbon source and achieved degradation efficiencies of 14.6% and 8.2%, respectively, after 48 h of incubation [39]. The relatively modest biotransformation observed here is consistent with the notion that microbial transformation of PFAS proceeds slowly under aerobic conditions, primarily due to the thermodynamic stability of the C–F bond [20], although detailed kinetic analyses would be required to fully elucidate this.

In both isolates, in addition to PFBS, two perfluorocarboxylates (PFCAs), namely PFBA and PFPrA, were detected as metabolites, with PFBS (70.5 mol%) and PFPrA (62.9 mol%) being the primary products in LH-1 and BO-1 treatments, respectively. This chain-shortening capacity observed in LH-1 and BO-1 stands in stark contrast to our previous findings with *Hyphomicrobium* sp. PF1, which degraded N-EtFOSA and PFOSA only to PFOS without detectable PFCA formation [41]. Meanwhile, the microbial community from Loring soil, an AFFF-contaminated soil collected from the former Loring Air Force Base (AFB; Aroostook County, ME), has been demonstrated to be capable of PFAS biotransformation but exhibits a limited biotransformation capacity toward PFOSA, as it can only convert PFOSA to PFOS without producing any detectable PFCAs during the entire degradation process [42]. These differences imply that the ability to truncate the perfluorinated carbon chain is not a universal feature of FASA-degrading bacteria but rather a strain-specific trait.

**Figure 7:**
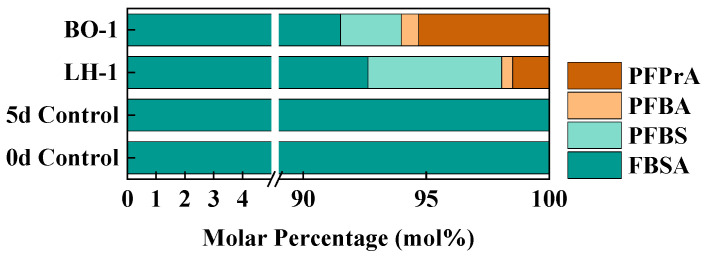
Molar percentage composition of FBSA and its degradation products in different treatment groups. Data are presented as mean values, *n* = 3.

As shown in Figure 7, PFBS, PFBA, and PFPrA were detected as degradation metabolites, with PFBS and PFPrA as the dominant products in the LH-1 and BO-1 groups, respectively, accounting for 5.44 mol% and 5.22 mol% of the total PFAS. The accumulation of PFBS as the dominant product in LH-1 indicates that this strain efficiently catalyzes the initial desulfonamidation of FBSA but has limited capacity for subsequent chain-shortening transformations. Similarly, *Pseudomonas aeruginosa* HJ4 degrades PFOS, yielding primarily PFBS and PFHxS as the main products, which are difficult to process further [16,41]. These comparisons suggest that the initial desulfonamidation step may be relatively widespread among diverse bacterial taxa, whereas the ability to proceed beyond PFBS (or PFOS in the CS system) requires additional enzymatic machinery that is not universally present. In contrast, the dominance of PFPrA in the BO-1 treatment indicates that this strain possesses the enzymatic capacity to carry out more extensive downstream transformations, including desulfonation and successive carbon-chain truncation steps. The formation of PFPrA, a C3 PFCA from the C4 precursor FBSA, implies at least one additional decarboxylation or *α*-oxidation step beyond PFBS formation. This deeper transformation capability of BO-1 is consistent with the genomic evidence discussed in Section 3.2, particularly the exclusive presence of fluorobenzoate degradation genes in this strain, which may provide enzymatic functions enabling perfluorinated carbon-chain processing [35]. Notably, a similar ability to generate both PFBA and PFPrA during PFAS degradation has been reported for Labrys portucalensis F11, which produces PFBA and PFPrA as downstream metabolites in the PFOS degradation pathway, consistent with the chain-shortening reactions observed in BO-1 [43]. The co-detection of PFBA as a minor product in both strains is also notable, as it may represent a transient intermediate between PFBS and PFPrA in the proposed pathway, although its low abundance precludes definitive assignment of its position in the transformation sequence based on endpoint data alone.

A rough mass balance analysis indicated that the sum of quantified products and residual FBSA was broadly consistent with the initial amount in each treatment, supporting the interpretation that biotransformation, rather than abiotic loss, was the primary driver of FBSA depletion. However, this mass balance should be regarded as approximate, as only targeted products were quantified and neither fluoride release nor additional untargeted intermediates were measured.

#### 3.3.2. Proposed Degradation Pathways and Genomic Corroboration

**Figure 8:**
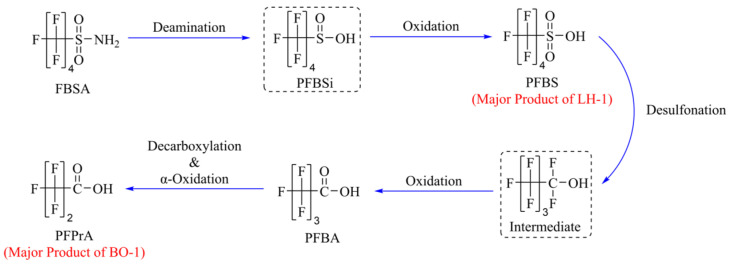
Proposed cascade biodegradation pathway of FBSA by strains LH-1 and BO-1. Compounds shown in dashed boxes were not quantitatively detected in this study.

Based on the detected metabolites and the genome annotations, Figure 8 summarizes a proposed FBSA biotransformation pathway for strains LH-1 and BO-1. The formation of PFBS in both cultures indicates that FBSA was transformed at the sulfonamide headgroup. This process is most likely initiated by desulfonamidation or hydrolytic deamination, during which the amino group of FBSA (C_4_F_9_SO_2_NH_2_) is substituted by an oxygen-containing group to yield PFBS (C_4_F_9_SO_3_H). Similar FBSA-to-PFBS transformation has been reported in several bacterial genera, including *Acinetobacter* [14], *Pseudomonas* [16], and *Gordonia* [19], suggesting that the cleavage or modification of the sulfonamide S–N bond is a more accessible microbial transformation step than defluorination of the perfluorinated carbon backbone [12,21]. Consistently, both strains harbor genes associated with hydrolysis and deamination reactions, including adenosine deaminase (*add*, *ADA*) and cytidine deaminase (*cdd*, *CDA*) (Table S5). However, because these genes are not specific markers for sulfonamide desulfonamidation, their role in FBSA transformation should be regarded as putative rather than functionally confirmed. Genes potentially involved in subsequent oxidative reactions are listed in Table S6. Collectively, these results suggest that FBSA transformation proceeds primarily through modification of the sulfonamide headgroup, followed by oxidation-related processes, although direct enzymatic evidence remains to be obtained.

Following the formation of PFBS, further transformation would require cleavage of the C–S bond and removal of the sulfonate group (–SO_3_^−^). This step is expected to be more difficult than the initial desulfonamidation of FBSA, because the C–S bond in perfluorinated sulfonates is relatively stable and less susceptible to enzymatic attacks [12]. In the proposed pathway, PFBS may first undergo desulfonation to generate a defluorinated or reactive perfluoroalkyl intermediate, which is subsequently oxidized to the corresponding perfluoroalkyl carboxylic acid. The detection of PFBA (C_3_F_7_COOH) and PFPrA (C_2_F_5_COOH) in both cultures supports the occurrence of downstream transformation and chain-shortening reactions after PFBS formation. Genome annotation further shows that both strains harbor genes associated with sulfur metabolism, including *ssuE* and *msuE*, which may be involved in reductive or oxidative reactions related to organosulfur compound transformation. These genes provide a possible genetic basis for PFBS desulfonation, although their direct roles in cleaving the C–S bond of perfluorinated sulfonates remain to be experimentally verified. Candidate genes potentially involved in desulfonation are summarized in Table S7. After desulfonation, the resulting C4 intermediate may be further oxidized to PFBA, which could then enter a sequential chain-shortening process. One possible route involves decarboxylation of PFBA to generate a C3 perfluorinated intermediate, followed by further oxidation to PFPrA. This proposed sequence is consistent with the detection of both PFBA and PFPrA in the cultures and resembles transformation patterns reported for *Gordonia* sp. strain NB4-1Y, which can degrade fluorotelomer sulfonamide alkyl carboxylates and 6:2 fluorotelomer sulfonate under sulfur-limited conditions [19]. Nevertheless, because intermediate compounds were not fully resolved and the responsible enzymes have not been directly characterized, this pathway should be interpreted as a putative transformation route rather than a confirmed biochemical sequence. Genes potentially associated with decarboxylation and defluorination are listed in Tables S8 and S9, respectively.

**Figure 9:**
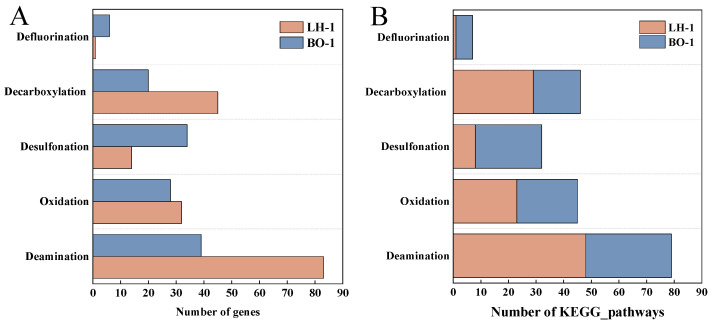
Functional gene and KEGG pathway annotations associated with the proposed FBSA biodegradation pathway in strains LH-1 and BO-1. (**A**) Number of functional genes potentially involved in key transformation processes, including defluorination, decarboxylation, desulfonation, oxidation, and deamination. (**B**) Number of KEGG pathways associated with these metabolic functions.

The comparison of gene counts and corresponding KEGG pathways associated with each proposed reaction step of the FBSA degradation pathway between strains LH-1 and BO-1 is shown in Figure 9. The divergent metabolite profiles of the two strains can be interpreted within this pathway framework. In strain LH-1, FBSA transformation appeared to be largely limited after the initial desulfonamidation step, as PFBS was the dominant product, whereas only minor amounts of downstream products, including PFBA and PFPrA, were detected. This pattern suggests that the subsequent desulfonation, oxidation, and chain-shortening steps may be genetically incomplete or functionally limited in LH-1 under the tested conditions. In contrast, strain BO-1 showed a greater capacity to proceed through the downstream transformation sequence, as indicated by the pronounced accumulation of PFPrA, suggesting more active PFBS desulfonation and subsequent chain-shortening reactions. This interpretation is consistent with the genomic evidence that BO-1 harbors a broader set of genes associated with xenobiotic degradation and organofluorine-related transformation pathways, including fluorobenzoate degradation genes (Section 3.2). Although these genes are primarily annotated for the degradation of fluorinated aromatic compounds [35], their presence may reflect a broader metabolic potential for processing fluorinated intermediates. Nevertheless, the specific roles of these candidate genes in FBSA degradation remain putative and require further validation through transcriptomic analysis, targeted enzyme assays, or genetic manipulation-based validation approaches.

### 3.4. Evaluation of Degradation Capability Toward PFOA and PFOS

**Figure 10:**
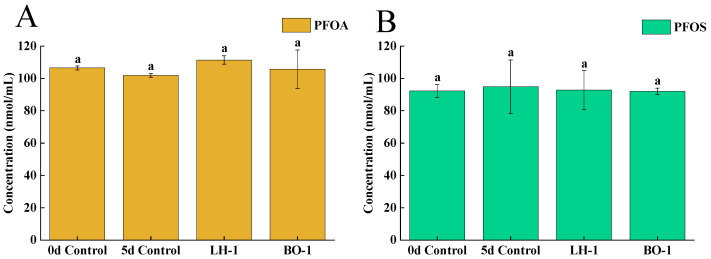
Residual molar concentration of PFOA (**A**) and PFOS in different treatment groups (**B**). Data are presented as mean ± SD (*n* = 3; *p* > 0.05). Different letters indicate significant differences (*p* < 0.05).

To examine substrate specificity under the same assay framework, strains LH-1 and BO-1 were further tested for their ability to transform PFOA and PFOS (Figure 10). Neither strain caused a statistically significant change in PFOA or PFOS concentrations relative to the abiotic controls during the 5-day incubation period (*p* > 0.05), indicating that no detectable degradation of either compound. This observation is consistent with our previous study on a precursor PFAS-degrading bacterium, PF1, which was able to transform PreFOSs but showed no detectable degradation of PFOS or PFOA [44]. Similarly, *Acinetobacter* sp. M has been reported to transform PFOSA into PFOA, whereas further conversion of PFOA was not observed [14].

Together, these findings highlight a fundamental distinction between precursor biotransformation and terminal PFAS degradation. Enzymatic reactions involved in precursor transformation commonly target relatively labile functional groups or non-fluorinated structural moieties, such as S–N, C–N, or C–H bonds, through processes including desulfonamidation, deacetylation, and oxidation. These reactive features are absent or much less accessible in fully fluorinated terminal products such as PFOA and PFOS. Therefore, substantial degradation or mineralization of PFOA and PFOS would ultimately require cleavage of highly stable C–F bonds [20], which remains a major challenge for aerobic microorganisms. To date, microbial defluorination of PFOA or PFOS has only been reported in a limited number of systems, typically under specialized redox conditions. For example, *Acidimicrobium* sp. strain A6 was reported to partially defluorinate PFOA and PFOS under iron-reducing conditions, using hydrogen or ammonium as the electron donor [17]. The lack of detectable PFOA and PFOS transformation by LH-1 and BO-1 under aerobic conditions therefore further supports the interpretation that these strains primarily mediate precursor transformation rather than degradation of terminal PFCAs.

## 4. Conclusions

Our study demonstrated that two newly isolated sediment bacteria, *Neobacillus* sp. LH-1 and *Glutamicibacter* sp. BO-1, could biodegrade sulfonamide-based PFAS precursor FBSA into PFBS, PFBA, and PFPrA. Both strains exhibited strict substrate specificity to-ward FBSA, achieving 5-day removal efficiencies of 8.78% and 11.37%, respectively, while showing no transformation of legacy PFOS and PFOA. Both strains shared an initial desulfonamidation step yielding PFBS, but their subsequent transformation pathways diverged markedly. LH-1 showed limited downstream processing and accumulated PFBS as the dominant product, whereas BO-1 exhibited greater capacity for further desulfonation and chain-shortening reactions, producing PFPrA as the major metabolite. Genome annotation further revealed strain-specific differences in genes associated with deamination, desulfonation, oxidation, decarboxylation, and defluorination, providing a putative genetic basis for their divergent transformation profiles. Both strains exhibited complementary transformation traits and robust tolerance to FBSA, suggesting their potential for tailored in situ bioremediation, though further optimization studies are needed before practical application. Overall, these findings provide a foundational basis for developing microbe-based remediation strategies targeting FBSA and related PFAS precursors in marine environments.

## Data Availability

Data are available in the article/Supplementary Materials and on request from the corresponding author.

## Supplementary Materials

The following supporting information can be downloaded at: https://www.mdpi.com/article/10.3390/toxics14070564/s1, Text S1. Bacteria draftmap method; Text S2. Sample treatments in microbial cultures for PFAS analysis; Text S3. Instrumental analysis; Text S4. Quality assurance and quality control; Text S5. Data analysis; Table S1. Information on the sampling site; Table S2. Liquid chromatography gradient elution conditions for the determination of the list of PFASs by UPLC-MS/MS; Table S3. List of PFASs monitored in the present study, the optimized UPLC-MS/MS parameters, the recoveries (%), the method detection limits (MDLs, 3:1 S/N) and the method quantification limits (MQLs, 10:1 S/N) in blank culture medium (pmol/mL); Table S4. Summary of Whole-Genome Sequencing (WGS) Statistics; Table S5. The genes related to deamination obtained through WGS of strains LH-1 and BO-1; Table S6. The genes related to oxidation obtained through WGS of strains LH-1 and BO-1; Table S7. The genes related to desulfonation obtained through WGS of strains LH-1 and BO-1; Table S8. The genes related to decarboxylation obtained through WGS sequencing of strains LH-1 and BO-1; Table S9. The genes related to defluorination obtained through WGS of strains LH-1 and BO-1; Figure S1. Growth curves of strains LH-1 and BO-1. The bacterial biomass was characterized by OD_600_ values, and the growth dynamics of the two strains within 170 h were recorded. References [28,45,46,47] are cited in Supplementary Materials.
