# Supplementary material for: Isolation and Characterization of Two Perfluorobutane Sulfonamide (FBSA)-Degrading Bacterial Strains, Neobacillus sp. LH-1 and Glutamicibacter sp. BO-1, from Estuarine and Marine Sediments
Source: Toxics. 2026 Jun 27;14(7):564. doi: 10.3390/toxics14070564 (PMC13418901; doi:10.3390/toxics14070564)
Supplement: Supplementary file 1 [file toxics-14-00564-s001.zip › toxics-4361247-supplementary.pdf]

Supporting Information

Isolation and Characterization of Two Perfluorobutane Sulfonamide (FBSA)-Degrading Bacterial Strains, *Neobacillus* sp. LH-1 and *Glutamicibacter* sp. BO-1, from Estuarine and Marine Sediments

Chenhe Zhao, Mengjin Feng, Kairui Wang, Yvyan Gao, Jiasong Zhao and Shuyan Zhao *

Key Laboratory of Industrial Ecology and Environmental Engineering, Ministry of Education, School of Chemical Engineering, Ocean and Life science, Dalian University of Technology, Panjin, Liaoning, P.R. China 124221

*Corresponding Authors: S.Y. Zhao (zhaoshuyan@dlut.edu.cn)

Total pages: 20

Number of tables: 9

Number of figures: 1

Contents

Text S1. Bacteria draftmap method 1

Text S2. Sample treatments in microbial cultures for PFAS analysis 1

Text S3. Instrumental analysis 2

Text S4. Quality assurance and quality control 2

Text S5. Data analysis 2

References 3

Table S1. Information on the sampling site 4

Table S2. Liquid chromatography gradient elution conditions for the determination of the list of PFAS by UPLC-MS/MS. 5

Table S3. List of PFAS monitored in the present study, the optimized UPLC-MS/MS parameters, the recoveries (%) , the method detection limits (MDLs, 3:1 S/N) and the method quantification limits(MQLs,10:1 S/N) in blank culture medium(pmol/mL). 6

Table S4. Summary of Whole-Genome Sequencing (WGS) Statistics. 7

Table S5. The genes related to deamination obtained through WGS sequencing of strains LH-1 and BO-1. 8

Table S6. The genes related to oxidation obtained through WGS sequencing of strains LH-1 and BO-1. 11

Table S7. The genes related to desulfonation obtained through WGS sequencing of strains LH-1 and BO-1. 13

Table S8. The genes related to decarboxylation obtained through WGS sequencing of strains LH-1 and BO-1. 15

Table S9. The genes related to defluorination obtained through WGS sequencing of strains LH-1 and BO-1. 17

Figure S1. Growth curves of strains LH-1 and BO-1. The bacterial biomass was characterized by OD_{600} values, and the growth dynamics of the two strains within 170 hours were recorded. 18

Text S1. Bacteria draftmap method

The experimental procedure mainly includes three stages: sample DNA processing, library construction, and on-machine sequencing. First, DNA quality control was performed on the samples. Genomic DNA was then randomly sheared into fragments of approximately 350 bp in length using a Covaris disruptor. After end repair, 3′-end A-tailing, and ligation of sequencing adapters, fragment size selection was conducted via a two-step method using the Agencourt SPRIselect kit. High-fidelity PCR amplification was subsequently applied to obtain a sufficient yield of the DNA library. The insert size of the library was examined using the Agilent 5400 system, and its effective concentration was accurately quantified by qPCR. Bridge PCR amplification was then carried out, followed by PE150 high-throughput sequencing on the Illumina NovaSeq platform (Lim et al., 2016). Initial denaturation at 95 °C for 5 min; 30 cycles of denaturation at 95 °C for 30 s, annealing at 55 °C for 30 s, and extension at 72 °C for 90 s, followed by a final extension step at 72 °C for 10 min. Stringent experimental conditions were maintained throughout the process to guarantee the quality of the library and the reliability of sequencing data.

Systematic bioinformatics analysis was performed after sequencing. Raw reads were initially filtered to remove low-quality reads, reads with excessive N bases, and reads contaminated by adapter sequences, yielding clean data for subsequent analysis. Genome assembly was conducted using multiple programs including SOAPdenovo (Li et al., 2008) and SPAdes (Bankevich et al., 2012). The assembled results were integrated, and gap closing was performed to generate the final assembly, from which short fragments below 500 bp were filtered out. Further genome component analysis was carried out: coding genes were predicted using GeneMarkS; repetitive sequences were identified by RepeatMasker and TRF; and non-coding RNAs, genomic islands, and other functional elements were annotated with corresponding bioinformatic tools. Finally, gene functional annotation was accomplished via sequence alignment against public databases including GO, KEGG, and COG.

Text S2. Sample treatments in microbial cultures for PFAS analysis

Sample treatments in microbial cultures were based on the method used in our previous study (Zhao et al., 2016).The sample preparation procedure was carried out as follows. A 1.0-mL portion of the exposure broth was taken and diluted to 50 mL using ultrapure water. This diluted solution was subjected to solid-phase extraction (SPE) on Cleanert HLB cartridges (500 mg/6 mL, Agela Technologies, China). Prior to sample loading, the cartridges were conditioned by passing 10 mL of methanol and then 10 mL of ultrapure water. The diluted sample was loaded at a flow rate of one drop per second. The cartridges were subsequently washed with 5 mL of ultrapure water and dried under vacuum. Target analytes were then eluted with 10 mL of methanol into 15-mL polypropylene centrifuge tubes. The eluates were concentrated to about 0.5 mL under a gentle nitrogen stream, then

reconstituted to 1.0 mL with ultrapure water to obtain a 1:1 (v/v) methanol/water mixture. The final extract was filtered through a 0.22-μm organic PTFE syringe filter and placed in amber autosampler vials for instrumental analysis.

Text S3. Instrumental analysis

The analysis of PFAS was conducted on a Waters UPLC system interfaced with a Waters XEVO-TQS tandem mass spectrometer (UPLC-MS/MS, Waters Corporation, Milford, MA, USA). A BEH C18 analytical column (1.7 μm, 2.1 mm × 50 mm, Waters) together with a guard column was used to resolve the analytes, with the column thermostat set to 38 °C. The mobile phases, consisting of 2 mmol/L ammonium acetate in water (solvent A) and methanol (solvent B), were pumped at a flow rate of 0.45 mL/min. A detailed gradient elution program is provided in Table S1. Detection was performed in negative electrospray ionization (ESI^-) mode using multiple reaction monitoring (MRM). The optimized instrument parameters included a capillary voltage of 2.2 kV, a desolvation temperature of 400 °C, a desolvation gas flow of 800 L/h, and a cone gas flow of 150 L/h. For all target PFAS, the specific MRM transitions, collision energies, and cone voltages are given in Table S2.

The field-emission scanning electron microscope (FE-SEM) used in this study is the Nova Nano SEM 450 manufactured by FEI Company, USA. This instrument supports both high-vacuum and low-vacuum imaging modes, and is furnished with a secondary electron (SE) detector, a backscattered electron (BSE) detector, and an infrared CCD camera. It operates with a landing voltage range of 3–5 kV and delivers a continuously variable magnification from 1× to 100,000×. For secondary electron imaging in high-vacuum mode, the nominal resolution reaches 1.0 nm at an accelerating voltage of 15 kV and 1.4 nm at 1 kV. The system is coupled with an EDAX energy-dispersive X-ray (EDX) spectrometer, which covers an elemental detection range from beryllium (Be, atomic number Z = 4) to uranium (U, atomic number Z = 92) with an energy resolution superior to 126 eV.

Text S4. Quality assurance and quality control

A systematic validation of the external standard quantification method was conducted to confirm its feasibility and accuracy, using matrix spike-and-recovery tests. In these tests, a precisely defined theoretical concentration of target mixed standards was added to uninoculated blank biological matrices. The resulting method detection limits (MDLs) and method quantification limits (MQLs) were calculated from the blank matrices at signal-to-noise ratios of 3:1 and 10:1, respectively. Table S2 provides the recoveries, MDLs, and MQLs for all analyzed PFAS.

Text S5. Data analysis

The degradation efficiency (E, %) was calculated as:

$$E(\%)=\frac{C_{0d}-C_{5d}}{C_{0d}}\times 100 \quad (1)$$

where C0d is the concentration (nmol/mL) of the parent compound in the abiotic control on day 0, and C5d is the residual concentration (nmol/mL) in the biotic treatments at day 5.

The molar mass balance (MB, %) was calculated using Equation (2) to track the conversion of parent compounds into products:

$$MB(\%)=\frac{M_{residual,parent}+\sum M_{metabolites}}{M_{initial,control}}\times 100 \quad (2)$$

where Mresidual,parent represents the molar concentration of the residual parent compound (in nmol/mL) after 5 days of degradation. ∑Mmetabolites refers to the molar concentrations (in nmol/mL) of various degradation metabolites (e.g., PFBS, FBSA, PFPrA). Minitial,control is the initial molar concentration of the parent compound spiked into the system at the start of the experiment.

References

Lim, H.J.; Lee, E.H.; Yoon, Y.; Chua, B.; Son, A. Portable lysis apparatus for rapid single-step DNA extraction of *Bacillus subtilis*. *J. Appl. Microbiol.* **2016**, 120, 379-387.

Li, R.Q.; Li, Y.R.; Kristiansen, K.; Wang, J. SOAP: short oligonucleotide alignment program. *Bioinformatics* **2008**, 24, 713-714.

Bankevich, A.; Nurk, S.; Antipov, D.; Gurevich, A.A.; Dvorkin, M.; Kulikov, A.S.; Lesin, V.M.; Nikolenko, S.I.; Pham, S.; Prjibelski, A.D.; et al. SPAdes: A New Genome Assembly Algorithm and Its Applications to Single-Cell Sequencing. *J. Comput. Biol.* **2012**, 19, 455-477.

Zhao, S.Y.; Ma, X.X.; Fang, S.H.; Zhu, L.Y. Behaviors of N-ethyl perfluorooctane sulfonamide ethanol (N-EtFOSE) in a soil-earthworm system: Transformation and bioaccumulationethanol (N-EtFOSE) in a soil-earthworm system: Transformation and bioaccumulation. *Sci. Total Environ.* **2016**, 554-555, 186-191.

Table S1. Information on the sampling site.

	Site	Longitude	Latitude
Liaohe Estuary	T1	122°08′28″E	40°40′40″N
	T2	122°07′24″E	40°39′52″N
	T3	122°06′02″E	40°39′57″N
Bohai Sea, China	T4	121°43′57″E	40°48′41″N
	T5	121°50′03″E	40°46′43″N
	T6	121°54′20″E	40°43′55″N

Table S2. Liquid chromatography gradient elution conditions for the determination of the list of PFAS by UPLC-MS/MS.

**Time（min）**	**Aqueous ammonium acetate (Solvent A)（%）**	**Methanol (Solvent B)（%）**
0.0	80	20
0.5	80	20
2.0	60	40
3.0	75	25
5.0	15	85
5.1	0	100
7.0	0	100
9.0	80	20

Table S3. List of PFAS monitored in the present study, the optimized UPLC-MS/MS parameters, the recoveries (%) , the method detection limits (MDLs, 3:1 S/N) and the method quantification limits(MQLs,10:1 S/N) in blank culture medium(pmol/mL).

Compound	Monitoring transition (m/z)	Cone voltage (V)	Collision voltage (V)	Blank culture medium		
				Recovery	MDL	MQL
FBSA	298.00→78.00	20	85.0	93.8±5.2	0.0021	0.0072
PFOA	413.00→369.00	10.0	15.0	104±3.6	0.0026	0.0092
PFOS	498.90→79.90	40.0	40.0	124±3.8	0.0032	0.0103
TFA	113.00→69.00	10.0	20.0	79.1±5.6	0.0065	0.0224
PFPrA	163.00→119.00	10.0	15.0	92.3±8	0.0066	0.0220
PFBA	213.00→169.00	8.0	18.0	111±6.3	0.0024	0.0085
PFPeA	263.00→219.00	8.0	19.0	97.9±6.1	0.0026	0.0083
PFHxA	313.00→269.00	9.0	12.0	94.9±3.6	0.0017	0.0052
PFHpA	363.00→319.00	10.0	16.0	90.9±3.3	0.0011	0.0026
PFHxS	399.00→119.00	30.0	40.0	73.1±0.1	0.0034	0.0115
PFBS	299.00→99.00	25.0	40.0	93.2±7.3	0.0024	0.0081

Table S4. Summary of Whole-Genome Sequencing (WGS) Statistics.

	**LH-1**	**BO-1**
Genome size (bp)	6,122,270	3,658,926
Gene number	6,385	3,390
Gene total length (bp)	5261,412	3,178,368
Gene average length (bp)	824	938
GC%	38.92	61.97
Repeat rate (%)	10.20	12.74
Max length (bp)	1,022,866	1,200,623
Min length (bp)	538	3,215
GIs number	12	13
Prophage number	25	10
CRISPR number	7	2
tRNA number	153	65
sRNA number	7	1

Table S5. The genes related to deamination obtained through WGS sequencing of strains LH-1 and BO-1.

**Step**	**LH-1**			**BO-1**		
	Kegg_geneID	Ko_id	Ko_name	Kegg_geneID	Ko_id	Ko_name
Deamination	baca:FAY30_18585	K19689	ampS, pepS, ampT	glu:F0M17_07635	K03100	lepB
	ndt:L1999_23125	K01271	pepQ	glu:F0M17_07650	K03100	lepB
	cfir:NAF01_19490	K02236	comC	gmy:XH9_00935	K01438	argE
	baca:FAY30_17635	K01448	amiABC	gmy:XH9_00655	K01256	pepN
	ndt:L1999_22155	K08303	prtC, trhP	glu:F0M17_08135	K01259	pip
	nmk:CHR53_20395	K08303	prtC, trhP	glu:F0M17_08415	K23980	tpdA
	nmk:CHR53_20265	K08602	pepF, pepB	gmy:XH9_17080	K01284	dcp
	baca:FAY30_16970	K01308	yqgT	gmy:XH9_17015	K03101	lspA
	nmk:CHR53_19690	K19303	mepH	gmy:XH9_16415	K01256	pepN
	ndt:L1999_21375	K01262	pepP	glu:F0M17_10255	K01255	CARP, pepA
	gst:HW35_03840	K01258	pepT	gmy:XH9_15190	K01265	map
	ndt:L1999_20920	K07258	dacC, dacA, dacD	glu:F0M17_10365	K03101	lspA
	ndt:L1999_20830	K07258	dacC, dacA, dacD	glu:F0M17_10475	K19223	lytF, cwlE
	ndt:L1999_20685	K01449	cwlJ, sleB	glu:F0M17_10820	K01262	pepP
	nmk:CHR53_18740	K01463	bshB1	glu:F0M17_11445	K01265	map
	baca:FAY30_15680	K01299	E3.4.17.19	gmy:XH9_13435	K01354	ptrB
	baca:FAY30_15290	K01438	argE	gmy:XH9_12880	K07260	vanY
	ppsc:EHS13_10420	K07260	vanY	gpr:JQN66_14295	K01438	argE
	baca:FAY30_23235	K01449	cwlJ, sleB	gmy:XH9_10910	K01262	pepP
	baca:FAY30_23235	K01449	cwlJ, sleB	glu:F0M17_14600	K12251	aguB
	baz:BAMTA208_01205	K01449	cwlJ, sleB	gmy:XH9_02985	K23518	MACROD, ymdB
	baci:B1NLA3E_10015	K22278	pgdA	gmy:XH9_03210	K03743	pncC
	baca:FAY30_10555	K01299	E3.4.17.19	gmy:XH9_03605	K01297	ldcA
	baca:FAY30_10415	K01269	yhfE	gmy:XH9_04060	K01265	map
	nmk:CHR53_25155	K07130	kynB	gmy:XH9_08970	K11206	NIT1, ybeM
	nmk:CHR53_24030	K01443	nagA, AMDHD2	glu:F0M17_16865	K01267	DNPEP
	acai:ISX45_10125	K01262	pepP	glu:F0M17_00290	K01426	E3.5.1.4, amiE
	bacq:DOE78_23340	K01448	amiABC	gmy:XH9_08180	K01304	pcp

baci:B1NLA3E_12145	K07260	vanY
bon:A361_24670	K00681	ggt
baca:FAY30_19805	K21472	lytH
ndt:L1999_24975	K01255	CARP, pepA
bon:A361_23145	K13280	SEC11, sipW
baca:FAY30_19335	K01449	cwlJ, sleB
beo:BEH_13095	K07130	kynB
nmk:CHR53_25035	K11206	NIT1, ybeM
pfri:L8956_20280	K01438	argE
baci:B1NLA3E_06980	K21471	cwlO
cgot:J1899_08415	K01449	cwlJ, sleB
baca:FAY30_08330	K05823	dapL
nmk:CHR53_09280	K06383	spoIIGA
baca:FAY30_08825	K03101	lspA
baca:FAY30_09115	K03100	lepB
baca:FAY30_09165	K01419	hslV, clpQ
ndt:L1999_08870	K03742	pncC
baca:FAY30_04375	K01258	pepT
bou:I5818_15205	K01447	xlyAB
ndt:L1999_27870	K19224	lytE, cwlF
nmk:CHR53_16950	K01265	map
ndt:L1999_16255	K08602	pepF, pepB
ndt:L1999_16465	K12410	npdA
baca:FAY30_13985	K01262	pepP
baca:FAY30_24510	K19220	cwlS
ndt:L1999_27125	K01448	amiABC
gst:HW35_09455	K22135	bshB2
baca:FAY30_25500	K01258	pepT
nmk:CHR53_07435	K01443	nagA, AMDHD2
ndt:L1999_05605	K01308	yqgT
ndt:L1999_06070	K03100	lepB
baca:FAY30_07670	K08602	pepF, pepB
gmy:XH9_08165	K07259	dacB
gmy:XH9_07635	K21471	cwlO
gmy:XH9_07385	K22278	pgdA
glu:F0M17_01295	K00681	ggt
glu:F0M17_06335	K01443	nagA, AMDHD2
glu:F0M17_06390	K08281	pncA
glu:F0M17_06710	K07260	vanY
gmy:XH9_04870	K02654	pilD, pppA
aai:AARI_07510	K18455	mca
glu:F0M17_02635	K01322	PREP
gmy:XH9_08045	K07386	pepO

glp:Glo7428_4957	K01426	E3.5.1.4, amiE
ndt:L1999_04625	K19689	ampS, pepS, ampT
baca:FAY30_05915	K01426	E3.5.1.4, amiE
pvw:HU752_027095	K18540	ramA
gst:HW35_13210	K01266	dmpA, dap
ndt:L1999_12765	K01449	cwlJ, sleB
pasa:BAOM_3399	K22278	pgdA
ndt:L1999_13515	K21613	scmP
baci:B1NLA3E_06980	K21471	cwlO
baci:B1NLA3E_11115	K01259	pip
pbut:DTO10_07205	K12251	aguB
ndt:L1999_04720	K01567	pdaA
nmk:CHR53_06155	K01265	map
cfir:NAF01_07690	K12410	npdA
bae:BATR1942_07230	K07259	dacB
ntr:B0W44_16800	K11206	NIT1, ybeM
nmk:CHR53_01040	K01448	amiABC
ndt:L1999_03635	K17733	cwlK
pbut:DTO10_07865	K01270	pepD
nmk:CHR53_25140	K01270	pepD
ndt:L1999_27635	K01448	amiABC
ndt:L1999_26630	K19220	cwlS
baca:FAY30_00075	K07258	dacC, dacA, dacD

Table S6. The genes related to oxidation obtained through WGS sequencing of strains LH-1 and BO-1.

Step	LH-1			BO-1		
	Kegg_geneID	Ko_id	Ko_name	Kegg_geneID	Ko_id	Ko_name
Oxidation	gaj:MY490_18915	K05913	dad	gmy:XH9_01245	K07104	catE
	baca:FAY30_14750	K00457	HPD, hppD	gmy:XH9_12105	K00529	hcaD
	baca:FAY30_14740	K00451	HGD, hmgA	glu:F0M17_13695	K00529	hcaD
	bon:A361_14510	K22443	cntA	glu:F0M17_13700	K05710	hcaC
	pasa:BAOM_3308	K05710	hcaC	gmy:XH9_11635	K15777	DODA
	prd:F7984_03855	K00483	hpaB	gpr:JQN66_14280	K00483	hpaB
	nmk:CHR53_04705	K05916	hmp, YHB1	glu:F0M17_13870	K03380	E1.14.13.7
	ndt:L1999_11475	K05916	hmp, YHB1	gmy:XH9_10580	K03380	E1.14.13.7
	bbad:K7T73_21240	K00483	hpaB	glu:F0M17_15605	K03380	E1.14.13.7
	baca:FAY30_13530	K21727	npcB	glu:F0M17_15620	K00529	hcaD
	nmk:CHR53_25160	K00453	TDO2, kynA	gmy:XH9_09300	K03381	catA
	adau:NZD86_23530	K06911	PIR	glu:F0M17_16125	K05784	benC-xylZ
	pnp:IJ22_49790	K15777	DODA	glu:F0M17_16130	K05550	benB-xylY
	anx:ACH33_04680	K22443	cntA	glu:F0M17_16135	K05549	benA-xylX
	bon:A361_08460	K08967	mtnD, mtnZ, ADI1	gmy:XH9_08795	K06911	PIR
	nmk:CHR53_12605	K00459	ncd2, npd	glu:F0M17_00430	K00459	ncd2, npd
	baca:FAY30_12925	K00459	ncd2, npd	gmy:XH9_07280	K20938	ladA
	ndt:L1999_16285	K15777	DODA	glu:F0M17_01345	K20938	ladA
	ndt:L1999_16820	K20940	phzS	glu:F0M17_01895	K00448	pcaG
	baca:FAY30_14685	K00459	ncd2, npd	glu:F0M17_01900	K00449	pcaH
	bfd:NCTC4823_00782	K16901	K16901	glu:F0M17_01905	K00481	pobA
	spoo:J3U78_11205	K00452	HAAO	glu:F0M17_05910	K03380	E1.14.13.7
	baqu:K6959_03030	K06911	PIR	aai:AARI_13280	K00446	dmpB, xylE
	ndt:L1999_03220	K00483	hpaB	glu:F0M17_03220	K00453	TDO2, kynA
	pbut:DTO10_25105	K15737	csiD	gmy:XH9_09735	K21481	mhuD
	ndt:L1999_09935	K22342	dmmA	glu:F0M17_15515	K21832	gbcB, bmoB
	ndt:L1999_09930	K22344	dmmC	gmy:XH9_09785	K00479	gbcA, bmoA
	ndt:L1999_09925	K22343	dmmB	glu:F0M17_02600	K05916	hmp, YHB1

lmac:I6G82_23700	K22270	nagX	
lmac:I6G82_23685	K00450	E1.13.11.4	
ndt:L1999_01780	K22443	cntA	
baca:FAY30_00435	K01633	folB	

Table S7. The genes related to desulfonation obtained through WGS sequencing of strains LH-1 and BO-1.

Step	LH-1			BO-1		
	Kegg_geneID	Ko_id	Ko_name	Kegg_geneID	Ko_id	Ko_name
Desulfonation	ndt:L1999_10795	K00299	ssuE, msuE	gmy:XH9_05600	K00299	ssuE, msuE
	bsx:C663_0759	K01560	E3.8.1.2	glu:F0M17_15610	K00128	ALDH
	bbad:K7T73_21280	K00128	ALDH	glu:F0M17_00265	K00128	ALDH
	nmk:CHR53_20300	K00128	ALDH	glu:F0M17_15500	K00148	fdhA
	nmk:CHR53_13760	K00128	ALDH	ary:ATC04_02535	K00128	ALDH
	ndt:L1999_16470	K04072	adhE	glu:F0M17_00680	K13953	adhP
	cfir:NAF01_12425	K00128	ALDH	glu:F0M17_07220	K00826	E2.6.1.42, ilvE
	athe:K3F53_08260	K13954	yiaY	gmy:XH9_00835	K00640	cysE
	ndt:L1999_26740	K00121	frmA, ADH5, adhC	gmy:XH9_00830	K01738	cysK
	btm:MC28_2020	K00128	ALDH	gmy:XH9_00110	K00789	metK, MAT
	aaco:K1I37_05170	K00001	E1.1.1.1, adh	gmy:XH9_16760	K01738	cysK
	bvj:I5776_05535	K00114	exaA	glu:F0M17_10000	K01011	TST, MPST, sseA
	bvj:I5776_10820	K00114	exaA	gmy:XH9_15145	K05810	LACC1, yfiH
	bbad:K7T73_16910	K00128	ALDH	gmy:XH9_13790	K00812	aspB
				glu:F0M17_11745	K00133	asd
				glu:F0M17_11820	K01739	metB
				glu:F0M17_11825	K01697	CBS
				glu:F0M17_05345	K07173	luxS
				gmy:XH9_03335	K01243	mtnN, mtn, pfs
				gmy:XH9_04185	K00641	metX
				glu:F0M17_04525	K01740	metY
				glu:F0M17_01230	K01740	metY
				gmy:XH9_07055	K00928	lysC
				rhs:A3Q41_01403	K00558	DNMT1, dcm
				gmy:XH9_02520	K01011	TST, MPST, sseA
				gmy:XH9_02460	K00003	hom
				gmy:XH9_05890	K00831	serC, PSAT1
				glu:F0M17_07135	K00058	serA, PHGDH

	glu:F0M17_02695	K01739	metB
	glu:F0M17_12845	K01752	E4.3.1.17, sdaA, sdaB, tdcG
	glu:F0M17_03600	K01739	metB
	gmy:XH9_05195	K01740	metY
	gmy:XH9_05210	K00549	metE
	gpr:JQN66_03645	K00549	metE

Table S8. The genes related to decarboxylation obtained through WGS sequencing of strains LH-1 and BO-1.

**Step**	**LH-1**			**BO-1**		
	Kegg_geneID	Ko_id	Ko_name	Kegg_geneID	Ko_id	Ko_name
Decarboxylation	baca:FAY30_18700	K01611	speD, AMD1	gmy:XH9_00780	K00249	ACADM, acd
	baca:FAY30_18225	K01611	speD, AMD1	glu:F0M17_08595	K13038	coaBC, dfp
	nmk:CHR53_21025	K13767	fadB	gmy:XH9_00085	K01591	pyrF
	nmk:CHR53_20245	K01613	psd, PISD	gmy:XH9_15485	K00632	fadA, fadI
	baca:FAY30_16285	K01586	lysA	gmy:XH9_14745	K01599	hemE, UROD
	nmk:CHR53_18710	K01579	panD	glu:F0M17_12075	K01715	crt
	jeo:JMA_08890	K00249	ACADM, acd	glu:F0M17_13135	K01692	paaF, echA
	bon:A361_14575	K01715	crt	glu:F0M17_13510	K01579	panD
	gaj:MY490_05180	K06446	DCAA	roa:Pd630_LPD04635	K06446	DCAA
	bon:A361_14555	K07246	ttuC, dmlA	glu:F0M17_05205	K25044	oleBC
	ndt:L1999_11775	K00249	ACADM, acd	glu:F0M17_04920	K11410	acdH
	gst:HW35_00105	K04720	cobD	gmy:XH9_04010	K13745	ddc
	nmk:CHR53_20285	K01715	crt	kvr:CIB50_0000178	K01715	crt
	nmk:CHR53_20310	K01692	paaF, echA	gmy:XH9_08850	K16840	hpxQ
	baci:B1NLA3E_07750	K01604	mmdA	glu:F0M17_00195	K01715	crt
	msem:GMB29_09695	K13932	mdcD	gmy:XH9_07595	K01692	paaF, echA
	msem:GMB29_09690	K13931	mdcC	glu:F0M17_01880	K01607	pcaC
	panc:E2636_02920	K13929	mdcA	glu:F0M17_06240	K01586	lysA
	msem:GMB29_09675	K13935	mdcH	glu:F0M17_06650	K05921	hpaG
	baca:FAY30_19920	K07516	fadN	gmi:NMP99_04220	K07246	ttuC, dmlA
	ndt:L1999_25205	K00632	fadA, fadI			
	msem:GMB29_14670	K01715	crt			
	bkw:BkAM31D_03030	K01715	crt			
	scia:HUG15_17700	K13485	PRHOXNB, URAD			
	kpul:GXN76_09800	K16838	pucL			
	baca:FAY30_08410	K01585	speA			
	nmk:CHR53_09390	K01591	pyrF			
	nmk:CHR53_09430	K13038	coaBC, dfp			

etm:CE91St48_30860	K01715	crt
adau:NZD86_18470	K00249	ACADM, acd
scia:HUG15_13120	K00249	ACADM, acd
nmk:CHR53_27215	K18244	mmgC
bou:I5818_04775	K03392	ACMSD
bou:I5818_04805	K01617	dmpH, xylI, nahK
ndt:L1999_06010	K01599	hemE, UROD
bsj:UP17_01700	K23248	oiaC
baca:FAY30_05705	K00632	fadA, fadI
mdg:K8L98_08115	K01715	crt
bts:Btus_2019	K16838	pucL
bcir:C2I06_06005	K13485	PRHOXNB, URAD
scia:HUG15_22310	K00249	ACADM, acd
cthu:HUR95_15115	K00632	fadA, fadI
cfir:NAF01_15950	K00249	ACADM, acd
ndt:L1999_15190	K01607	pcaC
ndt:L1999_01790	K07246	ttuC, dmlA

Table S9. The genes related to defluorination obtained through WGS sequencing of strains LH-1 and BO-1.

Step	LH-1			BO-1		
	Kegg_geneID	Ko_id	Ko_name	Kegg_geneID	Ko_id	Ko_name
Defluorination	bsx:C663_0759	K01560	E3.8.1.2	gmy:XH9_09300	K03381	catA
				glu:F0M17_16120	K05783	benD-xylL
				glu:F0M17_16125	K05784	benC-xylZ
				glu:F0M17_16130	K05550	benB-xylY
				glu:F0M17_16135	K05549	benA-xylX
				glu:F0M17_00180	K01061	E3.1.1.45

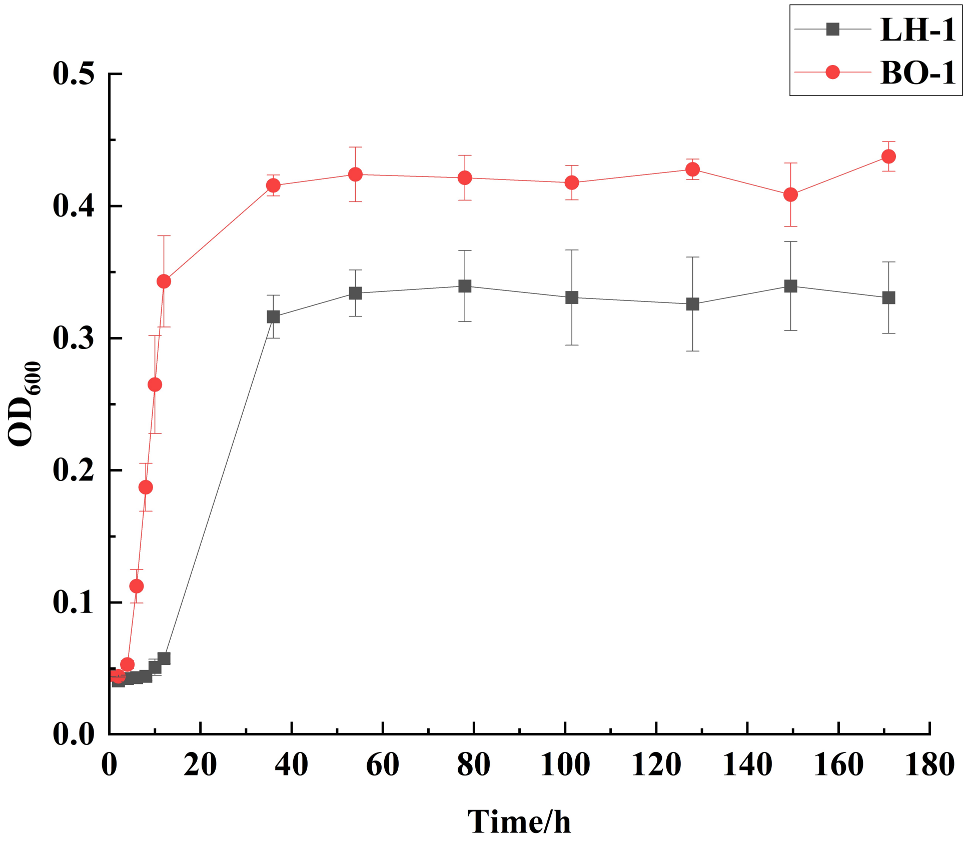

Figure S1. Growth curves of strains LH-1 and BO-1. The bacterial biomass was characterized by OD_{600} values, and the growth dynamics of the two strains within 170 h were recorded.
